# ID1 confers cancer cell chemoresistance through STAT3/ATF6-mediated induction of autophagy

**DOI:** 10.1038/s41419-020-2327-1

**Published:** 2020-02-20

**Authors:** Jiao Meng, Kaiyi Liu, Yang Shao, Xu Feng, Zhaodong Ji, Bin Chang, Yan Wang, Ling Xu, Gong Yang

**Affiliations:** 1 Cancer Institute, Fudan University Shanghai Cancer Center, Shanghai, 200032 China; 2 Department of Oncology, Shanghai Medical College, Fudan University, Shanghai, 200032 China; 3 Institute of Pediatrics, Children’s Hospital, Fudan University, Shanghai, 201102 China; 4 Department of Pathology, Fudan University Shanghai Cancer Center, Shanghai, 200032 China; 5 Department of Radiation Oncology, Fudan University Shanghai Cancer Center, Shanghai, 200032 China; 6 Department of Obstetrics and Gynecology, Minhang Hospital, Fudan University, Shanghai, 201199 China; 7 Central Laboratory, Shanghai Fifth People’s Hospital, Fudan University, Shanghai, 200140 China

**Keywords:** Autophagy, Cancer therapy

## Abstract

Chemoresistance is one of the major reasons leading to ovarian cancer high mortality and poor survival. Studies have shown that the alteration of cellular autophagy is associated with cancer cell chemoresistance. Here, we investigated whether the ovarian cancer chemoresistance is associated with the autophagy induced by the inhibitor of DNA binding 1 (ID1). By using gene overexpression or silencing, luciferase assay and human specimens, we show that ID1 induces high autophagy and confers cancer cell chemoresistance. The mechanistic study demonstrates that ID1 first activates the NF-κB signaling through facilitating the nuclear translocation of NF-κB p65, which strengthens the expression and secretion of IL-6 from cancer cells to subsequently activate the signal transducer and activator of transcription 3 (STAT3) through the protein phosphorylation at Y705. We further identified that STAT3 functions to promote the transcription of the activating transcription factor 6 (ATF6), which induces endoplasmic reticulum stress to promote cellular autophagy, granting cancer cell resistance to both cisplatin and paclitaxel treatment. Moreover, we found a significant correlation between the expression of ID1 and ATF6 in 1104 high grade serous ovarian cancer tissues, and that patients with the high expression of ID1 or ATF6 were resistant to platinum treatment and had the poor overall survival and progression-free survival. Thus, we have uncovered a mechanism in which ID1 confers cancer cell chemoresistance largely through the STAT3/ATF6-induced autophagy. The involved molecules, including ID1, STAT3, and ATF6, may have a potential to be targeted in combination with chemotherapeutic agents to improve ovarian cancer survival.

## Background

Epithelial ovarian cancer (EOC) is one of the most lethal gynecologic malignancies due to the lack of early diagnosis and chemoresistance. While both morbidity and mortality of this disease are rising every year^1,2^, the standard treatment for advanced disease is cytoreductive surgery and taxane/platinum-based chemotherapy^3,4^. Although taxol and cisplatin are the first-line chemotherapeutic agents to be used for ovarian cancer treatment, the resistance to these drugs has been a major obstacle to improve ovarian cancer survival^5^.

Inhibitor of DNA binding 1 (ID1) is a helix-loop-helix (HLH) protein that forms heterodimers with the members of the basic HLH family of transcription factors. The encoded protein has no DNA binding activity, but inhibits the DNA binding and transcriptional ability of the basic HLH proteins with which it interacts^6^. This protein may play a role in cell growth, senescence, and differentiation^7^ and is associated with ovarian cancer cell proliferation and apoptosis^8^. The expression of ID1 was positively correlated with poor differentiation, enhanced malignant potentiality, and more aggressive clinical behavior of epithelial ovarian tumor^9,10^. Although ID1 may promote chemoresistance through different pathways in different types of cancer, the clear mechanism is not fully understood in ovarian cancer chemoresistance^11–13^.

Autophagy is a highly conserved catabolic pathway in cells through degrading long-lived proteins and damaged organelles^14^. Autophagy plays important roles in various organismal processes, such as development, aging, and abnormalities, leading to various diseases including cancer^15^. Previous studies have demonstrated that autophagy is usually activated in cancer cells as a protective mechanism against numerous chemotherapeutic agents^16,17^. However, some studies reported that autophagy promotes chemosensitivity. For instance, the induction of autophagy by valproic acid enhances lymphoma cell chemosensitivity^18^, and RAD001 induces autophagy to promote the therapeutic response to cytotoxic chemotherapy of papillary thyroid cancer^19^. At present, whether ID1 induces autophagy to grant ovarian cancer cell chemoresistance is unknown. In this study, we have identified that ID1 confers ovarian cancer chemoresistance largely through the induction of the IL-6/STAT3/ATF6-mediated autophagy.

## Materials and methods

### Cell lines and cell culture

Human ovarian epithelial cancer cell lines (HEY, HEY A8, SKOV3, SKOV3 ip1, OVCA420, OVCA429, OVCA433, and A2780), and lentiviral packaging cells (HEK293T cells) were purchased from American Type Culture Collection (ATCC, USA) or maintained in our laboratory. All cell lines used in this study were identified for featured short tandem repeats (STRs) in public database by Suzhou Genetic Testing Biotechnology (Suzhou, China). All cell lines were subjected to routine test of mycoplasma contamination by using Universal Mycoplasma Detection Kit (ATCC® 30-1012K™) every 3 months during experiments. Cells were cultured with RPMI1640 or DMEM media supplemented with 10% fetal bovine serum, 100 U/mL penicillin, and 100 μg/mL streptomycin at 37 °C in a humidified 5% CO_2_ atmosphere.

### Plasmid construction, cell transfection, and viral infection

To enhance the expression of ID1, the human wild type cDNA of ID1 was cloned into pCDH-CMV-MCS-EF1-Puro lentiviral vector. Primers used for ID1 cDNA amplification were ID1-cDNA-FP and ID1-cDNA-RP (Table 1). The control vector used in this study was an empty lentiviral vector. To silence the expression of ID1, the DNA oligonucleotides were used to generate shRNAs against the open reading frames of ID1 mRNA. Oligonucleotides used for ID1 shRNA are shRNA-ID1-1, shRNA-ID1-2, and shRNA-ID1-3 (Table 1). The pLKO.1/puromycin ID1i was generated according to the protocol in website of http://www.addgene.org/tools/protocols/plko/. The control vector was similarly constructed by directly inserting a scrambled shRNA (Scr) into the pLKO.1/puromycin vector.

**Table 1:** Oligonucleotide sequences used in this study.

Primer name	Sequences		Purposes
ID1-cDNA-FP	5′-CGGAATTCATGAAAGTCGCCAGTGGCAGC-3′		ID1 cDNA plasmid construction
ID1-cDNA-RP	5′-CGGGATCCTCAGCGACACAAGATGCGATC-3′		ID1 cDNA plasmid construction
ID1-shRNA-1	5′-ATCGCATCTTGTGTCGCTGAA-3′		ID1 shRNA plasmid construction
ID1-shRNA-2	5′-CGACTACATCAGGGACCTTCA-3′		ID1 shRNA plasmid construction
ID1-shRNA-3	5′-CTACGACATGAACGGCTGTTA-3′		ID1 shRNA plasmid construction
ATF6 shRNA-1	5′-CCCAGAAGTTATCAAGACTTT-3′		ATF6 shRNA plasmid construction
ATF6 shRNA-2	5′- AAGTTGTGTCAGAGAACCAGA-3′		ATF6 shRNA plasmid construction
ATF6 shRNA-3	5′- AAGGAGGCACCTTCTAGGATT-3′		ATF6 shRNA plasmid construction
GAPDH-FP	5′-AGGTCGGTGTGAACGGATTTG-3′		qRT-PCR
GAPDH-RP	5′-TGTAGACCATGTAGTTGAGGTCA-3′		qRT-PCR
IL-6-FP	5′-CCTGAACCTTCCAAAGATGGC-3′		qRT-PCR
IL-6-RP	5′-TTCACCAGGCAAGTCTCCTCA-3		qRT-PCR
ATF6-1-FP	5′-AtatcGGTACCgtAGACTCGCTTGGACTTTGAC-3′		Promoter contruction (wild type)
ATF6-1-RP	5′-AttacCTCGAGctCCGTGATTAATATCTGGGAC-3′		Promoter contruction (wild type)
ATF6-2-FP	5′-AtatcGGTACCcaGTTGGAGTTCGTGATGTATG-3′		Promoter contruction (wild type)
ATF6-3-FP	5′-AtatcGGTACCagggtTCTGGGAAGCACATTTG-3′		Promoter contruction (wild type)
ATF6-4-FP	5′-AtatcGGTACCgtTACATCTGACGTAAGGGGA-3′		Promoter contruction (wild type)
ATF6-1-M-F	5′-GATAAACTTTGTggAGTCGAATTGATGTCTGCGTGTCTTCCCCCGCC-3′		Promoter contruction (mutant)
ATF6-1-M-R	5′-GGCGGGGGAAGACACGCAGACATCAATTCGACTccACAAAGTTTATC-3′		Promoter contruction (mutant)
ATF6-2-M-F	5′-GAGAATTATTCGTAAAAAggAAAGTAAATTTACTGTTAGTCTC-3′	Promoter contruction (mutant)	
ATF6-2-M-R	5′-GAGACTAACAGTAAATTTACTTTccTTTTTACGAATAATTCTC-3′	Promoter contruction (mutant)	
ATF6-3-M-F	5′-GCTTTGTTTCAATggATTTAAATAAAAGTAGTCTTTCTAGAAG-3′	Promoter contruction (mutant)	
ATF6-3-M-R	5′-CTTCTAGAAAGACTACTTTTATTTAAATccATTGAAACAAAGC-3′	Promoter contruction (mutant)	
ATF6-4-M-F	5′-GTATGTGATTTTCCTGTGATTTTCCTggAAATAAAACCCGAATC-3′	Promoter contruction (mutant)	
ATF6-4-M-R	5′-GATTCGGGTTTTATTTccAGGAAAATCACAGGAAAATCACATAC-3′	Promoter contruction (mutant)	

The lentiviral expression system was purchased from System Biosciences (SBI, USA). The plasmid was co-transfected with packaging vectors psPAX and pMD2.G at a ratio of 4:3:1.2 into HEK293T cells using Fugene HD (Promega, CA) for 48 h and harvested according to manufacturer’s instruction. The resulting supernatant was used to infect target cells with polybrene (10 μg/ml). Briefly, cells were infected twice for a total of 6 days (3 days for each infection) and the positive clones were selected with puromycin (1.0–2.5 μg/ml) or neomycin (200–800 μg/ml) for 10–14 days to establish new stable cell lines HEY-ID1 and HEY A8-ID1, OVCA429-ID1i and SKOV3 ip1-ID1i plus their controls according to the previously described protocols^20^.

ATF6-Myc-DDK-tagged cDNA and pCMV6-Entry (mammalian vector with C-terminal Myc-DDK Tag) were bought from OriGene (MD, USA). Oligonucleotides used for ATF6 shRNA is shRNA-ATF6-1, 2, and 3 (Table 1). ATF6 cDNA was introduced into OVCA429-ID1i and SKOV3 ip1-ID1i cells to generate OVCA429-ID1i-ATF6 and SKOV3 ip1-ID1i-ATF6 cell lines, respectively. All three ATF6 shRNA constructs were first validated for their effects on ATF6 silencing. We found that only ATF6 shRNA-1 was more effective (data not shown) than the others, so we introduced this shRNA construct into HEY-ID1 and HEY A8-ID1 cells to establish HEY-ID1-ATF6i-1 and HEY A8-ID1-ATF6i-1 cell lines, respectively. The control cell lines were constructed by using lentiviruses contain empty vector or scrambled shRNA as above.

### Cell treatment

In vitro cytotoxicity of cisplatin and paclitaxel was measured by MTT (Sigma-Aldrich) assay. Briefly, 5 × 10^3^ cells per well were plated into 96-well plates and treated with cisplatin (0, 0.4, 0.8, 1.6, 3.2, 6.4, 12.8, 25.6, 51.2, and 102.4 μM) or paclitaxel (0, 0.0625, 0.125, 0.25, 0.50, 1.0, 2.0, 4.0, 8.0, and 16 μM) or DMSO (diluent) for 48 h. Then, the medium with drugs or DMSO was replaced with 180 μL of fresh medium along with 20 μL of MTT solution (MTT dissolved in PBS at 5 mg/mL) in each well and incubated at 37 °C for 4 h. Last, the MTT-containing medium was discarded and 150 μL of DMSO per well was added to dissolve the newly formed formazan crystals. Absorbance of each well was determined by a microplate reader (Synergy H4, BioTek) at a 590-nm wavelength. Growth inhibition rates were calculated with the following equation, inhibition ratio = (OD_DMSO_−OD_drug_)/(OD_DMSO_−OD_blank_) × 100%.

Cells treated with the NF-κB inhibitor PS1145 (20 μM) for 4 or 8 h were used to detect the expression of IL-6 and other molecules by qPCR or by western blot. Cells treated with 1% of FBS (starvation), DMSO or chloroquine (CQ, 50 μM) at 4, 8, and 24 h were used to detect the expression of related proteins by immunofluorescence staining or western blot. Cells treated with IL-6 (30 ng/ml) for 4 h and/or S3I-201 (100 μM) for 24 h were used to detect the related protein expression or luciferase activities by western blot or luciferase assay.

### Cell proliferation, cell cycle, and apoptosis

Cell proliferation and cell cycle were determined by using the previously published methods^21^. To detect the drug-induced cellular apoptosis, cells were treated with cisplatin (3.5 μM) or paclitaxel (1.0 μM) or DMSO for 48 h. The cells were harvested, washed twice with cold 1 × PBS, and resuspended in 100 μL binding buffer at density of 1 × 10^5^ cells/mL. The cells were then stained with 5 μL 7AAD and Annexin V-PE (BD, USA) for 15 min in dark condition at room temperature and subjected to analysis by flow cytometer (Cytomics FC 500 MPL, Beckman Coulter, USA). The early apoptosis was evaluated based on the percentage of cells with Annexin V^+^/7AAD^−^, while the late apoptosis was that of cells with Annexin V^+^/7AAD^+^. The results were indicated as mean values from three independent determinations.

### Immunofluorescence staining

Immunofluorescence staining was done according to a published protocol^20^. Cells were treated with DMSO or chloroquine. Cells grown on cover slips were fixed in methanol for 5 min, permeabilized for 5 min with 0.3% Triton X-100 in PBS, and blocked for over 1 h with 5% bovine serum albumin and 1% goat serum. The cells were then incubated overnight at 4 °C with antibodies to ID1 (1:200), LC3B (1:200), ATF6 (1:100), or pSTAT3^705^ (1:100), followed by incubation with the secondary antibodies in a humid dark box at room temperature for 1 h. The secondary antibodies used were the FITC-conjugated donkey anti-mouse IgG, Cy3-conjugated donkey anti-rabbit IgG, Texas red-conjugated and FITC-conjugated donkey anti-goat IgG (Jackson ImmunoResearch Laboratory, USA). DAPI was obtained from Molecular Probes (USA). All stained cells were calculated and photographed with a Leica SP5 confocal fluorescence microscope (Leica Biosystems, GER).

### RNA isolation and real-time PCR

RNA was extracted from cell lines with Trizol and transcribed into cDNA using PrimeScript RT reagent Kit (TaKaRa, Japan). cDNAs were quantified by SYBR Premix Ex Taq (TaKaRa, Japan) by ABI 7500 fast real-time PCR System (Applied Biosystems, Carlsbad, USA). The expression level of IL-6 in cell lines was determined by quantitative real-time PCR (qRT-PCR). GAPDH mRNA was used as internal controls for normalization. Real-time PCR primers used for IL-6 and GAPDH were IL-6-FP, IL-6-RP, GAPDH-FP, and GAPDH-RP (Table 1).

### Transfecton of small interfering RNA

A rescue experiment was performed by knocking down of IL-6 using IL-6 siRNA in ID1 overexpression cell lines. IL-6 small interfering RNA (sc-39627) and control siRNA (sc-37007) were purchased from Santa Cruz Technology (California, USA). Transfection was conducted via Hieff Trans^TM^ Liposomal Transfection Reagent (YEASEN, Shanghai, China) according to the manufacture’s instruction. siRNAs used for transfection were at the concentration of 200 pmoL in 60-mm dishes. Cells were harvested and prepared to detect various proteins by western blot after 72 h of transfection.

### Immunohistochemistry staining

Ovarian normal and cancer tissues were obtained from the tissue bank of Fudan University Shanghai Cancer Center (FUSCC). This study was approved by the Clinical Research Ethics Committee of Fudan University Shanghai Cancer Center and conducted with an informed consent signed by each participants before the use of tissues (No. 1711178-23) according to the institutional guidelines. For immunohistochemistry (IHC), human or xenograft tumor samples were fixed in 10% formalin and embedded in paraffin wax. Unstained 3-mm sections were then cut from the paraffin blocks for IHC analysis. The sections were stained overnight at 4 °C with anti-ID1 (1:200), anti-LC3B (1:200), and anti-ATF6 (1:200). The secondary antibodies against mouse or rabbit IgG were obtained from an IHC kit, EnVision™ Detection Kit (GENE, USA). Diaminobenzidine (DAB) was used for coloration, and the color with dark brown was considered to be the strong positive staining.

### Western blot

To analyze protein expression in cells, western blot analysis was carried out according to standard methods. Antibodies against the following proteins were obtained from Santa Cruz Technology (California, USA): ID1 (sc-488), STAT3 (sc-8019), pSTAT3 (Tyr705, sc-8059), pSTAT3 (Ser 727, sc-8001), ATF6 (sc-166659), Beclin1 (sc-11427), and TFIIB (sc-274). Antibodies against the following proteins were obtained from Cell Signaling Technology (Massachusetts, USA): LC3B (cs-2775), p4E-BP1 (Ser65, cs-9451), 4E-BP1 (cs-9452). Antibodies against NF-ΚB p65 (610869) was obtained from BD Biosciences (San Jose, CA). β-actin (A2228, Sigma-Aldrich, USA) or β-tubulin (66240-1-Ig, Proteintech, USA) was detected as loading controls. The secondary antibodies anti-rabbit (cs-7074) and anti-mouse (cs-7076) were obtained from Cell Signaling Technology (USA). Immunoblotting reagents were from an electrochemiluminescence kit (Millipore, USA). All blots were exposed for visualization between 5 s and 2 min. The intensity of protein bands were quantified by Image J software (http://imagej.nih.gov/ij/download.html) to calculate the ratios of IntDen (Proteins)/IntDen (β-actin, β-tubulin, or TFIIB) to ensure that the detection of protein bands was linearized.

### Luciferase reporter assay

The primers used to construct the full-length and truncated promoters of ATF6 were listed in Table 1. The promoters containing putative 1, 2, 3, and 4 STAT3-binding sites were generated as pGL3-ATF6-1, pGL3-ATF6-2, pGL3-ATF6-3, and pGL3-ATF6-4. For dual-luciferase reporter assays, SKOV3 ip1 cells at a density of 1–2 × 10^4^ cells/well were seeded in 96-well plates and cultured for 24 h. The cells were co-transfected with STAT3 constitutive activation (CA) or the expression control vector (200 μg/well), different ATF6 luciferase constructs or the control vector pGL3-basic (200 μg/well), and the control Renilla luciferase reporter vector pRL-TK (10 ng/well) (Promega, USA). After 48 h, luciferase assays were performed by the Dual-Glo® Luciferase Assay System (Promega, USA) and detected by a SynergyHT Multi-Mode Microplate Reader (BioTek, USA) at 595 nm. Site-specific mutagenesis was performed by Mut Express® II Fast Mutagenesis Kit (c212) (Vazyme, CHN). Mutant primers were designed by the guidance of the kit. All primers used to generate four mutant constructs were ATF6-1-M-FP, ATF6-1-M-RP, ATF6-2-M-FP, ATF6-2-M-RP, ATF6-3-M-FP, ATF6-3-M-RP, ATF6-4-M-FP, and ATF6-4-M-RP listed in Table 1. STAT3 CA and mutant ATF6 promoter constructs were co-transfected into cells using the above described method. Cells either treated with the STAT3 inhibitor S3I-201 (100 μM) and/or IL-6 (30 ng/ml) for 24 and 4 h, respectively, were also tested for the luciferase activity of ATF6 promoters.

### Xenograft tumors in nude mice

To detect the in vivo effects of ID1 on ovarian cancer, we selected the series of ovarian cancer cell lines to generate xenograft mouse tumor models. The animal experiments were approved by the Institutional Animal Care and Use Committee of Fudan University Shanghai Cancer Center and performed following the Institutional Guidelines and Protocols. Animals were purchased from Shanghai Slac Laboratory Animal Co. Ltd, housed in a specific pathogen free facility (Department of Laboratory Animals, Fudan University) and checked every 3 days. Six mice were used for each cell line. Briefly, 5 × 10^6^ or 1 × 10^7^ cells of HEY vector, HEY-ID1, HEY A8 vector, HEY A8-ID1, and SKOV3 ip1-Scr and SKOV3 ip1-ID1i cells were subcutaneously or intraperitoneally injected into 6-week-old BALB/c female athymic nude mice.

For subcutaneously injected animals, the date at which the first grossly visible tumor appeared was recorded, and tumor size was measured every 3 days thereafter. The longest diameter “*a*” and the shortest diameter “*b*” of tumors were measured and the tumor volume was calculated with the use of the following formula: tumor volume (in mm^3^) = *a* × *b*^2^ × 0.52. When the biggest tumor in any mouse reached 20 mm in diameter, all experimental mice were sacrificed simultaneously and the tumor sizes were measured accordingly. Mice given i.p. injections were observed for lethargy, poor appetite, and abdominal enlargement, and euthanasia was performed with poor survival situations. The metastatic nodules derived from liver, omentum, mesentery, and lower pelvic were collected for statistical analysis. Tumors were removed and fixed in 10% formalin overnight and subjected to routine histological and immunohistochemical examination after being embedded and sectioned^22^.

### Dataset analysis

A dataset of microarray gene expression consisting of 1104 serous ovarian cancer cases treated with platinum was downloaded from The Cancer Genome Atlas (TCGA, http:tcga-data.nci.nih.gov) and assessed. The gene expression levels were provided as log2 ratios. The analysis of Spearman’s correlation was performed using GraphPad Prism software. Patient survivals were analyzed by the Kaplan–Meier software using the online tool KM-plotter (http://kmplot.com/analysis). An auto-selected best cutoff was performed using online software.

### Statistical analysis

The data were calculated using GraphPad Prism software and expressed as the mean ± S.E. Comparisons between control and treated groups were determined by two-sided *t* test. Multiple comparisons were not performed. *P* < 0.05 is considered statistically significant (*P < 0.05; **P < 0.01; ***P < 0.001). Center values are mean, and error bars are S.D.

## Results

### ID1 promotes ovarian cancer tumor growth

**Fig. 1:**
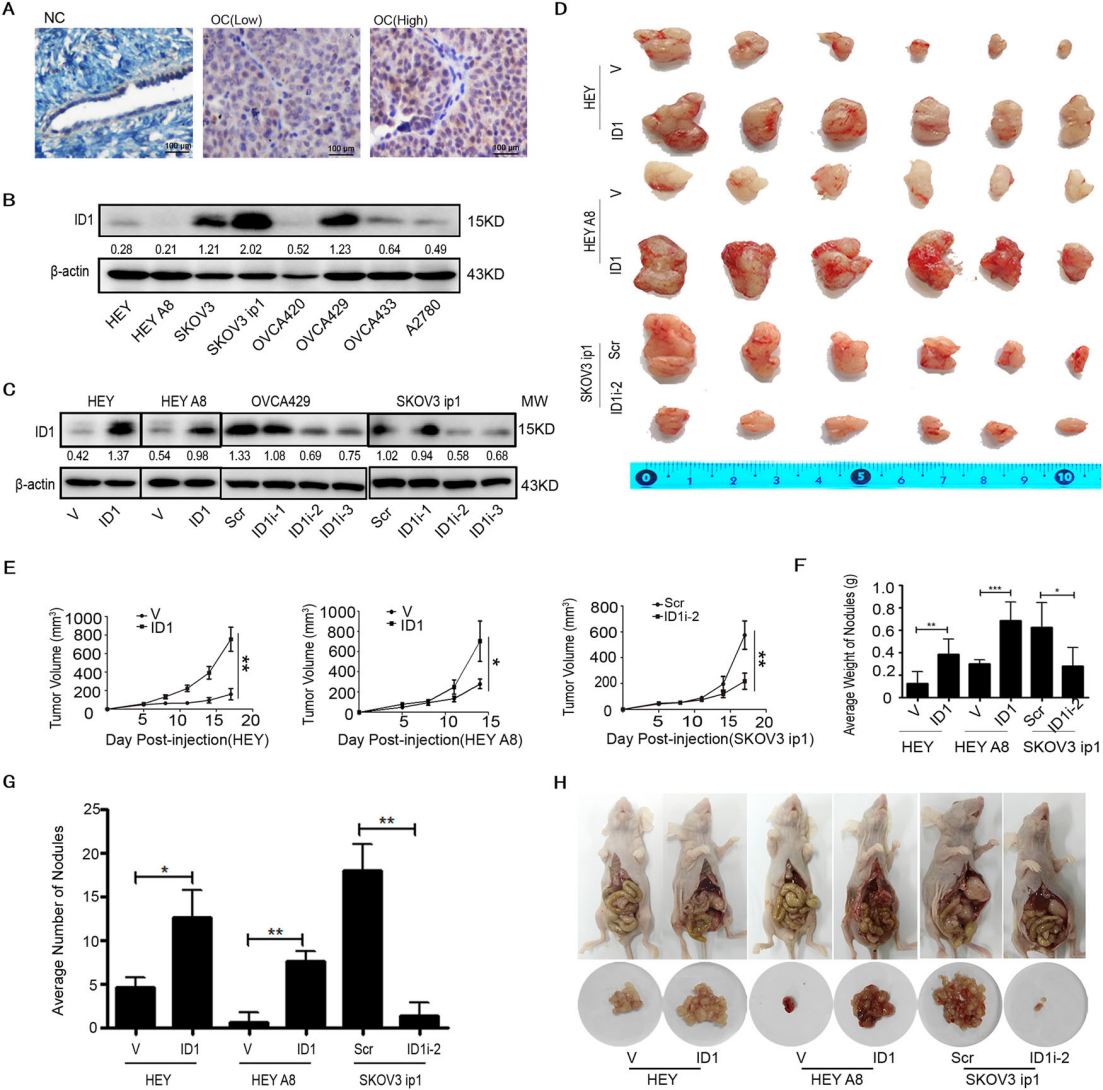
Tumor growth and metastasis induced by ID1. **a** Differences of ID1 expression detected by IHC in representative ovarian normal and cancer tissues. NC stands for normal control; OC stands for ovarian cancer. **b** Analysis of ID1 expression by western blot in eight ovarian cancer cell lines. **c** Examination of ID1 expression in ID1 overexpression or silencing cells by western blot. **d**, **e** Tumor tissues isolated from mice subcutaneously injected with cells expressing ID1 cDNA or shRNA (**d**), and tumor growth curves (**e**). **f**, **g** Average weight (F) and number (G) of the nodules dissected from peritoneal injection mice. **h** Animals with peritoneal tumor and nodules dissected from liver, omentum, mesentery, and lower pelvic. Representative images are shown. V stands for vector. ID1 stands for ID1 cDNA; Scr stands for scrambled shRNA; ID1i stands for ID1 shRNA. All error bars = 95% CIs. *P < 0.05, **P < 0.01, ***P < 0.001. β-actin was used as a loading control.

To investigate the function of ID1 in ovarian cancer, we first detected the expression level of ID1 in 6 normal ovarian or 21 cancer tissues, and found that no ID1 was detected in all normal tissues and high nuclear ID1 expression was in 15 (71.4%) cancer tissues (Fig. 1a). Two cases appeared with weak cytoplasmic and nuclear expression of ID1 (data not shown). In eight ovarian cancer cell lines, low ID1 was detected by western blot in HEY, HEY A8, OVCA420, OVCA433, and A2780 cells, while high expression of ID1 was conceived in SKOV3, SKOV3 ip1, and OVCA429 cells (Fig. 1b). Therefore, we overexpressed ID1 in HEY and HEY A8 cells, and silenced the expression of ID1 in SKOV3 ip1 and OVCA429 cells. Consequently, ID1 was remarkably overexpressed or silenced in cells treated with ID1 cDNA (ID1) or ID1 shRNA (ID1i) compared with control cells treated with empty vector (V) or scrambled shRNA (Scr) (Fig. 1c).

Since other reports have indicated that ID1 induces cell proliferation and cell cycle alteration^23,24^, we performed a limited study. The results showed that cell proliferation was promoted by ID1 overexpression but inhibited by ID1 silencing (SFig. 1A). Cell population at G0/G1 phase was significantly decreased or increased by ID overexpression or silencing, whereas cell population at S phase was inversely altered by ID1 overexpression or silencing (SFig. 1B-C).

To confirm the biological function of ID1 in ovarian cancer cells, the tumor growth rate was validated by subcutaneous implantation of cells into female BALB/c-nude mice. Compared with controls, cells with overexpression of ID1 enhanced the tumor growth, whereas cells with knockdown of ID1 retarded the growth of tumor (Fig. 1d, e).

To determine whether ID1 contributes to ovarian cancer metastasis in vivo, we performed animal assays by intraperitoneal injection of above cells. The average weights and number of metastatic nodules derived from liver, omentum, mesentery, and lower pelvic were significantly higher or lower in xenografts animals injected with ID1 overexpression or silencing cells than in those injected with control cells (Fig. 1f–h). These data suggest that ID1 promotes ovarian cancer tumor growth and metastasis.

### ID1 confers ovarian cancer cell chemoresistance

**Fig. 2:**
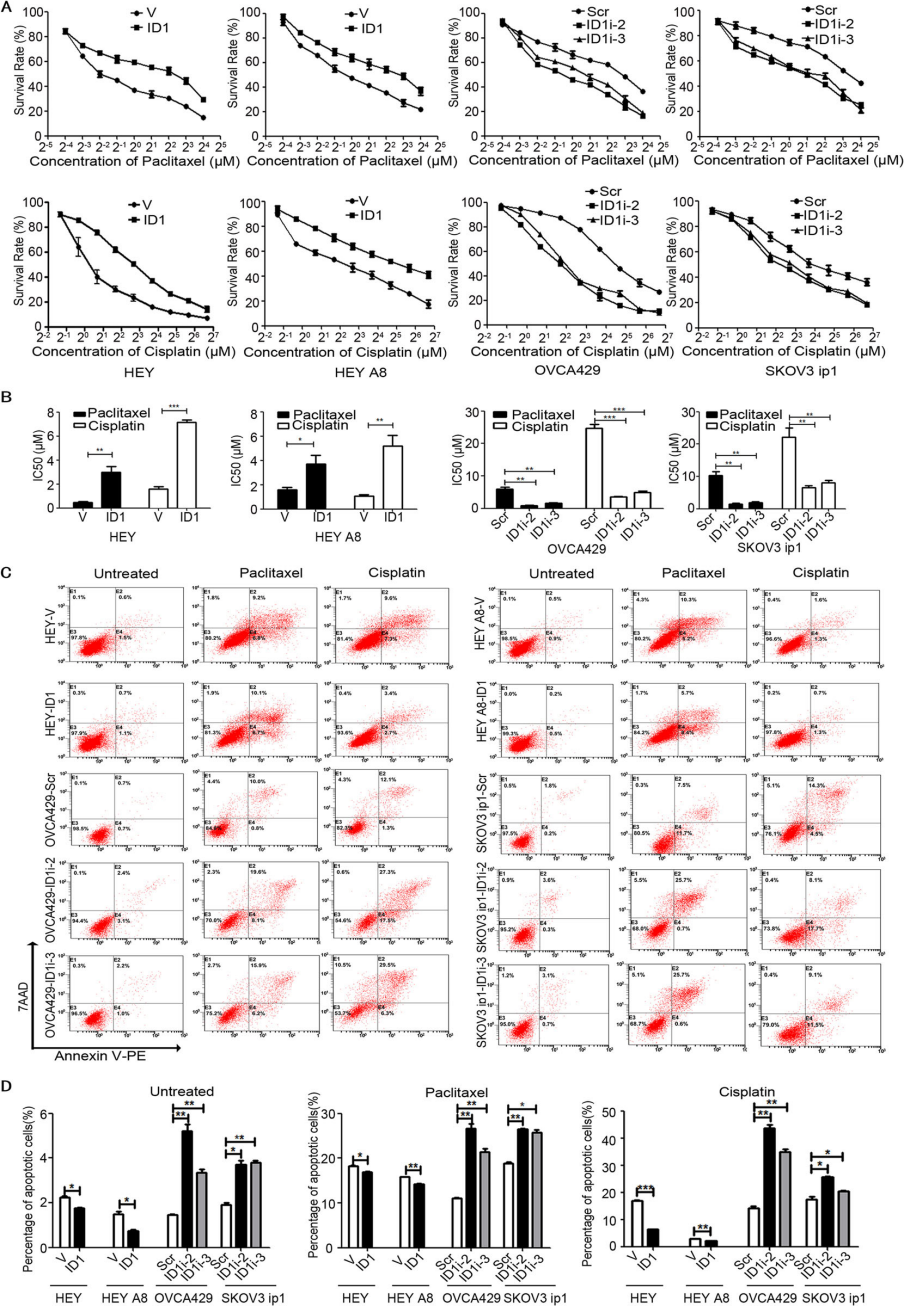
Treatment of cells with cisplatin and paclitaxel. **a** Survival rates of ovarian cancer cells treated with cisplatin and paclitaxel by MTT assay. OD values were tested after treatment of cisplatin and paclitaxel at 48 h. **b** IC50 values of ovarian cancer cells with or without ID1 cDNA or shRNA against cisplatin and paclitaxel treatment. Bars represent SD from three independent experiments. **c** Representative apoptotic profiles induced by cisplatin (3.5 μM) and paclitaxel (1.0 μM) tested by flow cytometry. **d** Apoptosis quantification by flow cytometer. Representative diagrams and quantification are shown. Bars represent SD from three independent experiments. All error bars = 95% CIs. *P < 0.05, **P < 0.01, ***P < 0.001.

To explore the impact of ID1 on chemoresistance, cells were treated with different concentrations of cisplatin or paclitaxel for 48 h, and the cell viability was determined by MTT assay. As shown in Fig. 2, treatment of cells with cisplatin or paclitaxel resulted in a corresponding decrease of cell viability in a dose-dependent manner. The survival rate of cells overexpressing ID1 was increased, whereas that of cells expressing ID1 shRNA was decreased, compared with that of control cells (Fig. 2a). The IC50 values of cisplatin or paclitaxel were increased in cells overexpressing ID1. In contrast, the IC50 values in ID1-knockdown cells were highly reduced. Compared with the corresponding control cells, the IC50 values of paclitaxel were increased to 6-fold or 2-fold in ID1 overexpression HEY or HEY A8 cells and reduced to 7-fold or 5-fold in ID1-knockdown OVCA429 or SKOV3-ip1 cells, whereas the IC50 values of cisplatin were increased to 4-fold or 5-fold in ID1 overexpression HEY or HEY A8 cells and reduced to 7-fold or 3-fold in ID1-knockdown OVCA429 or SKOV3-ip1 cells, respectively (Fig. 2b). Specifically, the IC50 values of cisplatin and paclitaxel were 1.57 and 0.46 μM in HEY-V cells, but were 6.94 and 2.93 μM in HEY-ID1 cells, respectively. The IC50 values of cisplatin and paclitaxel were 21.7 and 10.19 μM in SKOV3ip1-Scr cells, but were 5.55 and 1.5 μM in SKOV3ip1-ID1i-2 cells, and 8.02 and 1.92 μM in SKOV3ip1-ID1i-3 cells (Fig. 2b). These data suggest that ID1 confers cisplatin and paclitaxel resistance in ovarian cancer cells.

To validate these results, cells treated with cisplatin or paclitaxel were double stained with Annexin-V/PE and analyzed by flow cytometer. The results showed that cisplatin or paclitaxel dramatically increased the percentage of cell apoptosis regardless of ID1 status. However, the apoptosis induced by cisplatin or paclitaxel was increased in cells transfected with ID1 shRNA, but decreased in cells overexpressing ID1 cDNA, compared with control cells (Fig. 2c, d). Specifically, the percentages of cell apoptosis were 2.3 and 1.7 in untreated HEY-V and HEY-ID1 cells, but were 18.3 or 17.1 in paclitaxel- or cisplatin-treated HEY-V cells, and 16.1 or 6.9 in paclitaxel- or cisplatin-treated HEY-ID1 cells, respectively. The percentages of cell apoptosis were 1.9, 3.7, and 3.8 in untreated SKOV3ip1-Scr, SKOV3ip1-ID1i-2, and SKOV3ip1-ID1i-3 cells, but were 19.2 or 18.4, 26.4 or 25.8, and 26.3 or 20.6 in paclitaxel- or cisplatin-treated cells, respectively (Fig. 2d). These results indicate that ID1 confers ovarian cancer cell chemoresistance.

### ID1 activates ATF6 to promote autophagy

**Fig. 3:**
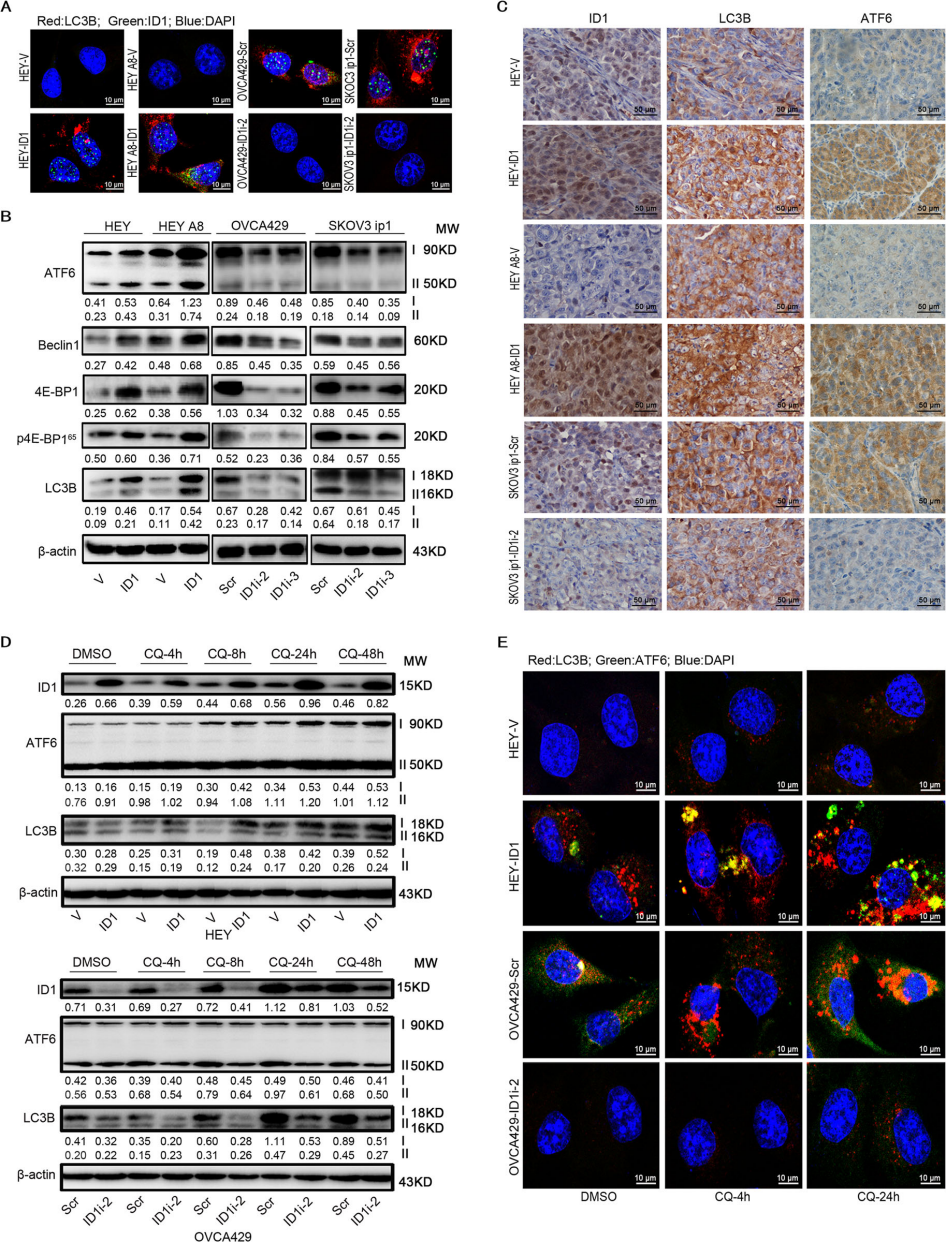
ID1 induces autophagy. **a** Foci of LC3B detected in ID1 overexpression and silencing cells by immunofluorescence. Red: LC3B; green: ID1; blue: DAPI. **b** Analysis of ATF6, Beclin1, 4E-BP1, p4E-BP1, and LC3B expression by western blot in cells transfected with ID1 cDNA or shRNA. **c** Detection of ID1, ATF6, and LC3B expression in xenograft tumor tissues by IHC. **d** Analysis of ID1, ATF6, and LC3B expression in HEY and OVCA429 cells treated with DMSO (diluent) or CQ (50 μM, 4, 8, 24, and 48 h). β-actin is used as a loading control. **e** Immunofluorescent images of LC3B and ATF6 in cells treated with DMSO (diluent) or CQ (50 μM, 4 and 24 h). Red: LC3B; green: ATF6; blue: DAPI.

To explore the mechanism of ID1 that induces chemoresistance, we first detected the cellular autophagy because the recent studies have shown that cancer cell chemoresistance may be associated with autophagy^25,26^. We found that the ectopic overexpresion of ID1 increased the formation of LC3B granule foci and silencing of ID1 inhibited this effect (Fig. 3a). We further used western blot to detect the alteration of the autophagy-related proteins. The results showed that the expression of ATF6, Beclin1, 4E-BP1, p4E-BP1, and LC3B was upregulated in ID1 overexpressing HEY and HEY A8 cells while their expression was downregulated in ID1 knocking down OVCA429 and SKOV3 cells, compared with control cells (Fig. 3b). To validate this result, we analyzed the expression of ID1, LC3B, and ATF6 in xenograft tumor tissues derived from previously conducted animal experiments. We found that the expression of LC3B and ATF6 appeared to be positively correlated with ID1 expression in xenograft tumor tissues (Fig. 3c). The levels of LC3B and ATF6 were increased in xenograft tumor tissues from animals injected with HEY-ID1 and HEY A8-ID1 but were decreased in SKOV3 ip1-ID1i cells, compared with control tissues (Fig. 3c). These data provide a clue that ATF6, an ER stress indicator, may be involved in the ID1-induced autophagy and chemoreristance.

Chloroquine (CQ) inhibits the activity of lysosome activity and arrests the latter step of autophagy and degradation of the autolysosome^27^. So we treated cells with CQ for 4, 8, 24, and 48 h and detected the expression LC3B and ATF6 by western blot and immunofluorescence. We found that the level of LC3B was accumulated in a time-dependent manner in both HEY-ID1 and OVCA429-Scr cells. The level of LC3B was increased or decreased obviously by CQ treatment in ID1 overexpression or knockdown cells. The level of ATF6 was increased or decreased in cells with ID1 overexpression or silencing. ATF6 was increased by ID1 overexpression and by CQ treatment. Surprisingly, we found that the full-length of ATF6 (upper band) was increased by CQ treatment particularly after 24 h in ID1 overexpressing HEY cells, whereas the cleaved ATF6 (lower band) was reduced by CQ treatment in ID1 silencing OVCA429 cells (Fig. 3d), although this phenomenon has never been reported by others. Compared with the corresponding control cells, the number of LC3B foci was obviously increased in cells overexpressing ID1, and decreased in cells with low levels of ID1 compared with control cells after CQ treatment (Fig. 3e). However, no obvious differences of LC3B were found between 24 and 48 h of treatment by CQ in both ID1 overexpressing or silencing cells. This may be due to that the alteration of LC3B reaches the limit at 24 h under the experimental concentration of chloroquine (CQ). These data indicate that the ID1-regulated ATF6 is independent of CQ treatment. CQ may be an ER stress inducer to promote the cleavage of ATF6, and ATF6 may mediate the ID1-induced autophagy.

### ID1 activates STAT3 through NF-κB-mediated upregulation of IL-6

**Fig. 4:**
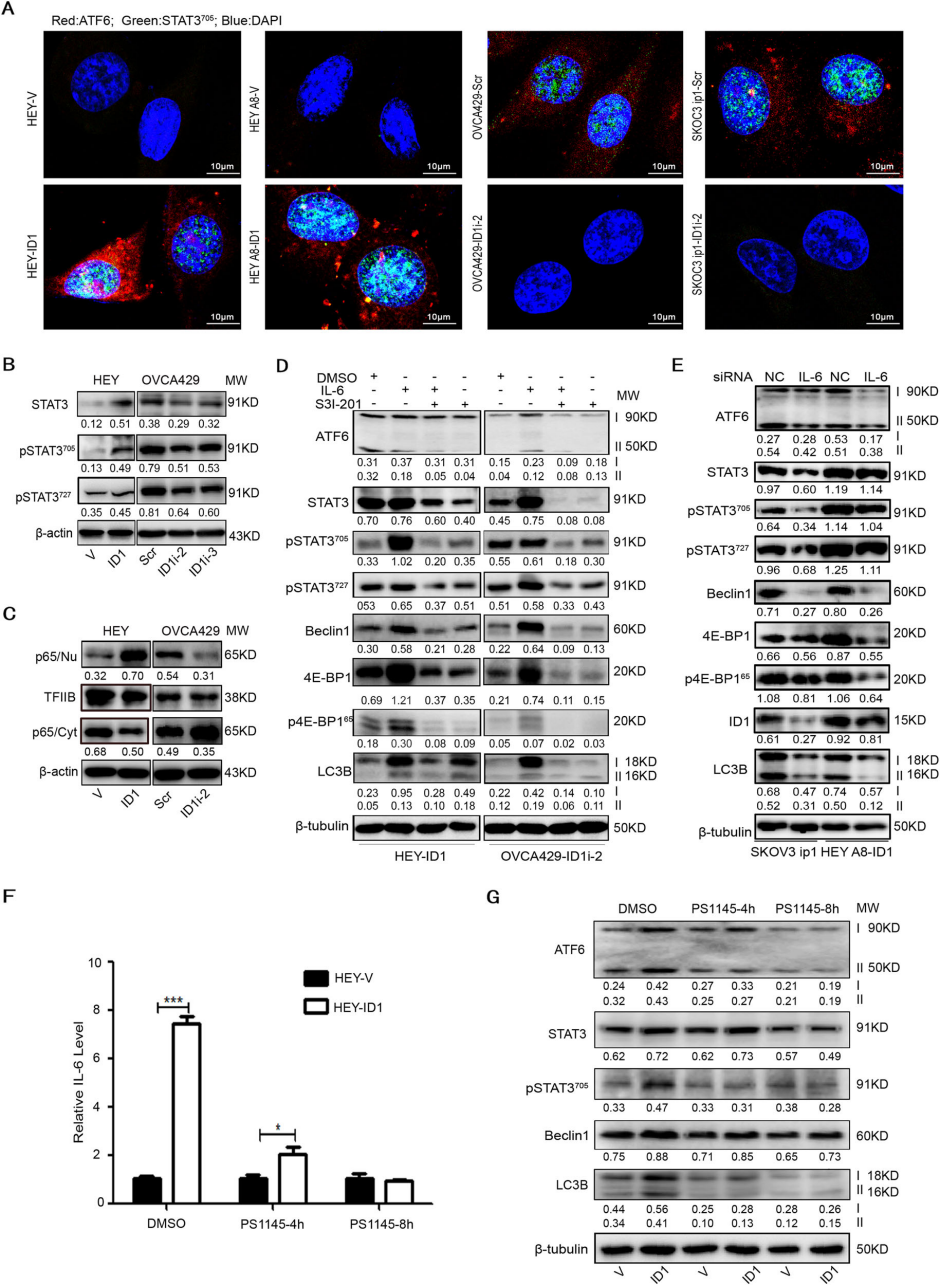
ID1 activates STAT3. **a** Immunofluorescent images of ATF6 and pSTAT3 (Y705) in ID1 overexpression cells. Red: ATF6; green: STAT3^705^; blue: DAPI. **b** Detection of the phosphorylated STAT3 in ID1 overexpression and silencing cells. **c** Detection of autophagy-associated proteins in ID1 overexpression and silencing cells treated with or without IL-6 and/or S3I-201. DMSO is diluent. β-actin and β-tubulin are used as loading controls. **d** Analysis of nuclear p65 and cytoplasmic p65 in ID1 overexpression and silencing cells. TFIIB is used as a nuclear loading control and β-actin is used as a cytoplasmic loading control. **e** Analysis of STAT3 phosphorylation and the autophagy-associated proteins in IL-6 knockdown cells. **f** Analysis of IL-6 level in HEY-ID1 cells treated with DMSO or PS1145 for 4 and 8 h by qRT-PCR. Bars represent SD from three independent experiments. All error bars = 95% CIs. *P < 0.05, **P < 0.01, ***P < 0.001. **g** Analysis of autophagy-related proteins in HEY-ID1 cells treated with or without PS1145 for 4 and 8 h. β-tubulin is a loading control.

Studies have shown that ID1 enhances angiogenesis via the PI3K/Akt and NF-κB/MMP-2 signaling pathways^28^, and that the ID1-mediated NF-κB activation is due to its physical interaction with p65^29^. Thus, we analyzed the STAT3 and NF-κB status in ID1 overexpression or knockdown cells. The results showed that ID1 overexpression or silencing mainly induced or reduced the phosphorylation of STAT3 at Tyr705 (Fig. 4a, b). Overexpression of ID1 led to the increased nuclear accumulation of NF-κB p65 and a decreased cytosolic level of NF-κB p65, whereas knockdown of ID1 reduced or enhanced the p65 protein levels in nucleus or the cytoplasm of OVCA429 cells (Fig. 4c). Because STAT3 may be activated by IL-6^30^, we treated cells with IL-6 at the concentration of 30 ng/mL for 4 h or with STAT3 inhibitor S3I-201 at the concentration of 100 μM for 24 h. We found that IL-6 activated the STAT3 phosphorylation and increased the expression of the autophagy-associated proteins. In contrast, inhibition of STAT3 by S3I-201 reduced the expression of both STAT3 and pSTAT3, as wells as the autophagy-associated molecules (Fig. 4d). To further investigate if IL-6 plays a critical role in the ID1-induced autophagy, we silenced the expression of IL-6 in SKOV3 ip1 and HEY A8-ID1 cell lines by using IL-6 siRNA. Disruption of IL-6 downregulated the expression of both STAT3 and pSTAT3 in ID1 high expression cells, which resulted in the downregulation of the autophagy-associated proteins including LC3B (Fig. 4e). Interestingly, we also observed a downregulation of ID1 (Fig. 4e). Because PS1145 is an IκB kinase (IKK) inhibitor and inhibits IL-6 secretion^31^, we treated cells overexpressing ID1 with or without PS1145. The results showed that PS1145 treatment reduced the expression of IL-6 (Fig. 4f). Although PS1145 treatment inhibited the expression of ATF6, STAT3, pSTAT3, Beclin1, and LC3B in both vector control and ID1 overexpressing cells, the expression of these proteins was more inhibited by PS1145 at 4 h in vector cells than in ID1 overexpressing cells (Fig. 4g). However, the treatment with PS1145 at 8 h equally inhibited the expression of these proteins in both control and ID1 expressing cell lines.

These results indicate that ID1 promotes STAT3 phosphorylation through the NF-κB-mediated upregulation of IL-6, and that STAT3 may play an important role in ID1-induced autophagy.

### ATF6 mediates the ID1/STAT3-induced autophagy

**Fig. 5:**
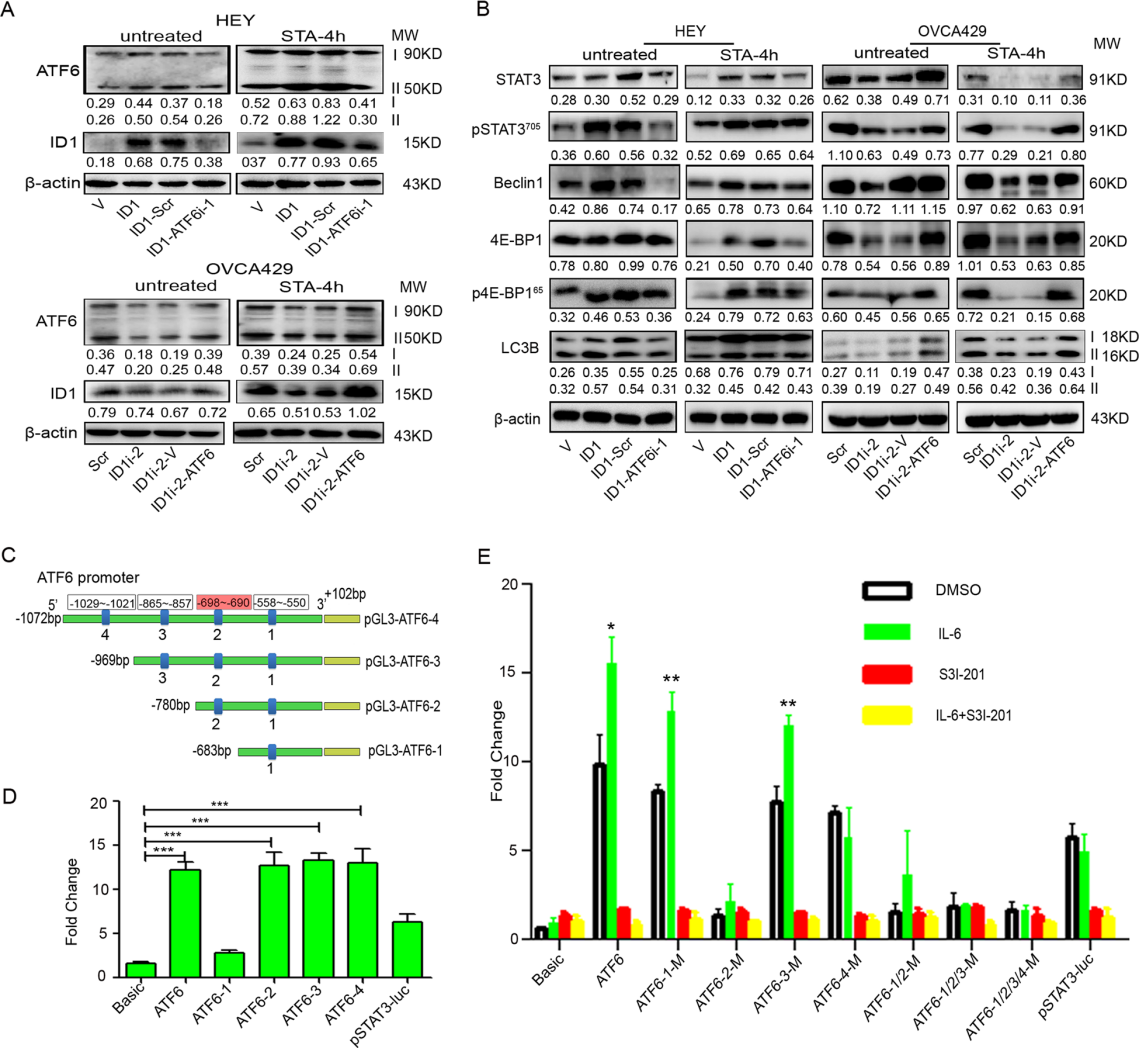
ATF6 mediates the ID1-induced autophagy and is transactivated by STAT3. **a** Effects of ATF6 and cell starvation on the expression of ID1 and ATF6. **b** Alteration of STAT3, pSTAT3, ATF6, Beclin1, 4E-BP1, p4E-BP1, and LC3B in ID1, and ATF6 overexpression or silencing cells treated with or without starvation for 4 h. β-actin is a loading control. **c** A schematic diagram showing the STAT3-binding sites in ATF6 promoter constructs for luciferase assays. Blue boxes and numbers indicate the binding sites and sequences. **d** Dual-luciferase reporter assay tested in SKOV3 ip1 cells transiently co-transfected with STAT3, Renilla luciferase, and various ATF6 promoter fragment constructs as indicated. Bars represent SD from three independent experiments. **e** STAT3 activates ATF6 promoter transcription. IL-6 (30 ng/ml) activated STAT3 and magnified the luciferase activity while S3I-201 (50 μM) inhibits STAT3 and reduces the luciferase activity of ATF6 transcription. Bars represent SD from three independent experiments. All error bars = 95% CIs. *P < 0.05, **P < 0.01, ***P < 0.001.

Autophagy is rapidly induced to high levels by starvation as an ER stress to promote the degradation of intracellular components to support metabolism in the absence of extracellular nutrients^32^. In starvation, autophagy may provide a nutrient source to maintain survival^33^. To analyze the role of ATF6 in ID1-induced autophagy, we first silenced or overexpressed ATF6 in ID1 overexpression or knockdown cells and treated the resulting cells and parental cells with starvation (STA). We observed that silencing of ATF6 in ID1 overexpressing HEY cells downregulated the expression of ID1 in both control and starved cells (Fig. 5a upper panel), and that overexpression of ATF6 in ID1 silencing OVCA429 cells upregulated the ID1 levels, particularly in starved cells (Fig. 5a lower panel). Moreover, knockdown of ATF6 inhibited the expression of STAT3, pSTAT3, Beclin1, 4E-BP1, p4E-BP1, and LC3B while overexpression of ATF6 increased the expression of these proteins, although treatment of ATF6 shRNA expressing HEY-ID1 cells with starvation did not clearly downregulate the expression of STAT3, pSTAT3, Beclin1, p4E-BP1, and LC3B. These results suggest that ID1 and ATF6 have a synergistic effect on autophagy through the regulation of STAT3 activation (Fig. 5b).

To investigate whether STAT3 potentially regulates the expression of ATF6 at the transcription level, we analyzed the promoter region of ATF6 from −969 to +102 bp, and found four consensus STAT3-binding motifs (TTMXXXDAA, D = A/G; M = A/C; X = any) in the ATF6 promoter. Therefore, we inferred that STAT3 may positively regulate the expression of ATF6 through transcription activation. To determine the binding activity of ATF6 promoter with STAT3, a full-length and three truncated ATF6 promoters containing 4, 3, 2, or 1 STAT3 potential binding sites were cloned into pGL3-basic vector to yield four pGL3-ATF6 recombinant constructs: pGL3-ATF6-4, pGL3-ATF6-3, pGL3-ATF6-2, and pGL3-ATF6-1 (Fig. 5c). The results from the luciferase assay performed in SKOV3 ip1 cells showed that the constructs of pGL3-ATF6-2, pGL3-ATF6-3, and pGL3-ATF6-4 increased the luciferase activity, but pGL3-ATF6-1 remained the luciferase activity similar to the control, indicating that STAT3 is able to promote the transcription of ATF6 by binding to the promoter region at −969 to −580 nucleotides (Fig. 5d).

To determine which binding sites played the main role in ATF6 transcription, we constructed the mutants of pGL3-ATF6-1, pGL3-ATF6-2, pGL3-ATF6-3, and pGL3-ATF6-4 via PCR-directed mutagenesis. The results showed that the luciferase activity in SKOV3 ip1 cells was largely inhibited by transfection of cells with pGL3-ATF6-2-M compared with wild type or other mutant-transfected cells. Thus, the core sequence of the ATF6 promoter is located at pGL3-AFT6-2 containing the second STAT3-binding site, whereas the fourth STAT3-binding site may also have some minor affinities to bind to STAT3 protein. Furthermore, IL-6 magnified the luciferase activity while S3I-201 reduced the luciferase activity of ATF6 transcription (Fig. 5e). These data suggest that STAT3 promotes the transcription of ATF6 and the core sequence of ATF6-2 in the promoter region may play a major role in ATF6 transcription.

### ATF6 mediates the ID1-induced chemoresistance

**Fig. 6:**
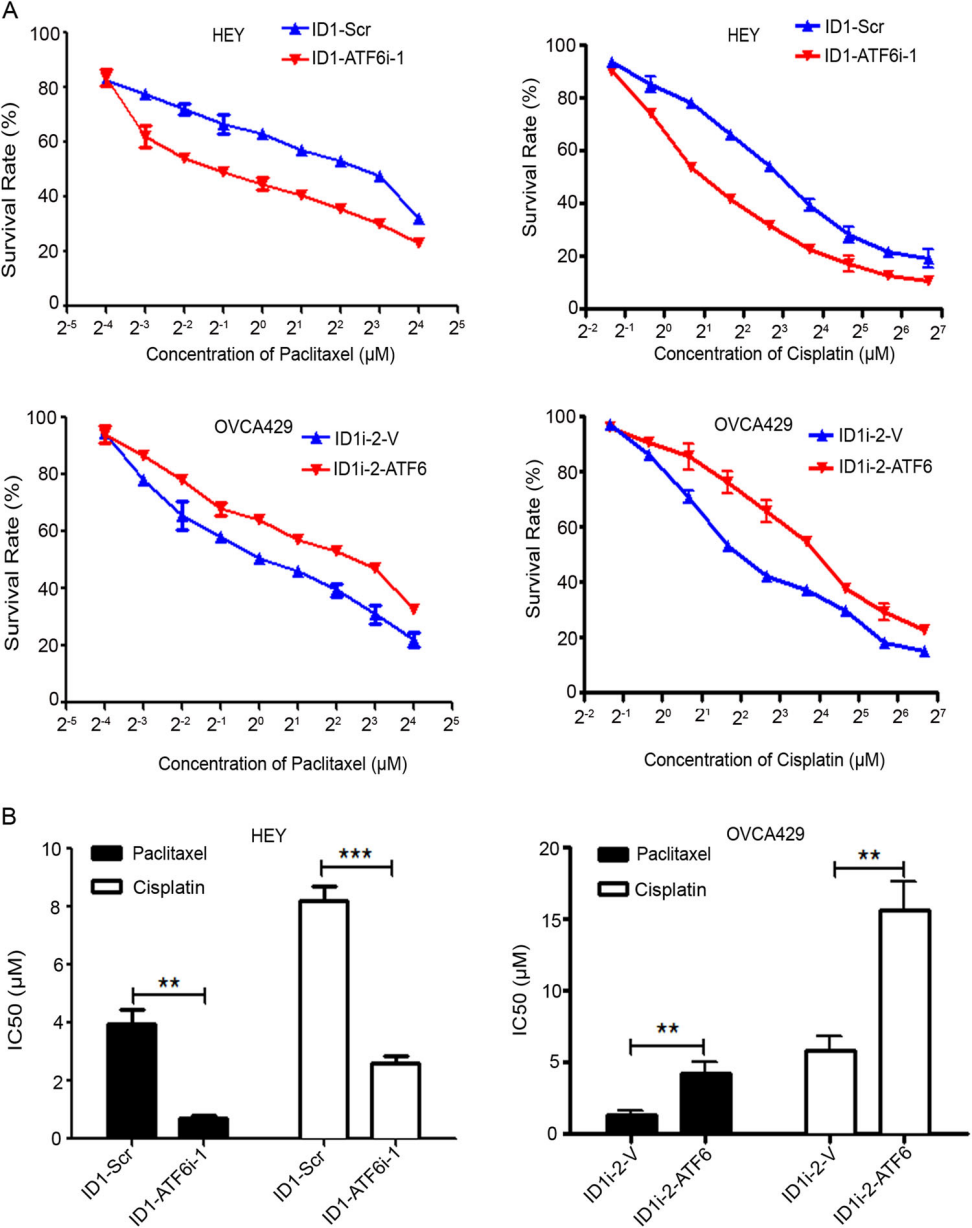
ID1 induces cancer cells to chemoresistance and ATF6 rescues this effect. **a** Survival rates of HEY and OVCA429 cells expressing cDNA/shRNA of ID1/ATF6 after treatment with paclitaxel and cisplatin by MTT assay. OD values were tested after treatment of cisplatin and paclitaxel 48 h. **b** IC50 values of cells against paclitaxel and cisplatin treatment. Bars represent SD from three independent experiments. All error bars = 95% CIs. *P < 0.05, **P < 0.01, ***P < 0.001.

Since the ectopic expression of ID1 increased the resistance of cells to paclitaxel or cisplatin. These results are consistent with those reported in various references^34–36^. More experiments were designed to detect whether the alteration of ATF6 could change the sensitization of ovarian cancer cells to chemotherapeutic agents. We used paclitaxel or cisplatin to treat ATF6 silencing or overexpression cells that were formerly introduced with ID1 cDNA or shRNA. The results showed that interruption of ATF6 reduced the IC50 values in ID1 overexpressing cells, whereas overexpression of ATF6 lifted the IC50 values in ID1 silencing cells, compared with control cells. These results demonstrate a synergistic effect of ID1 and ATF6 on the chemoresistance of ovarian cancer cells (Fig. 6a, b).

### ID1 and ATF6 predicts poor survival for platinum resistant ovarian cancer patients

**Fig. 7:**
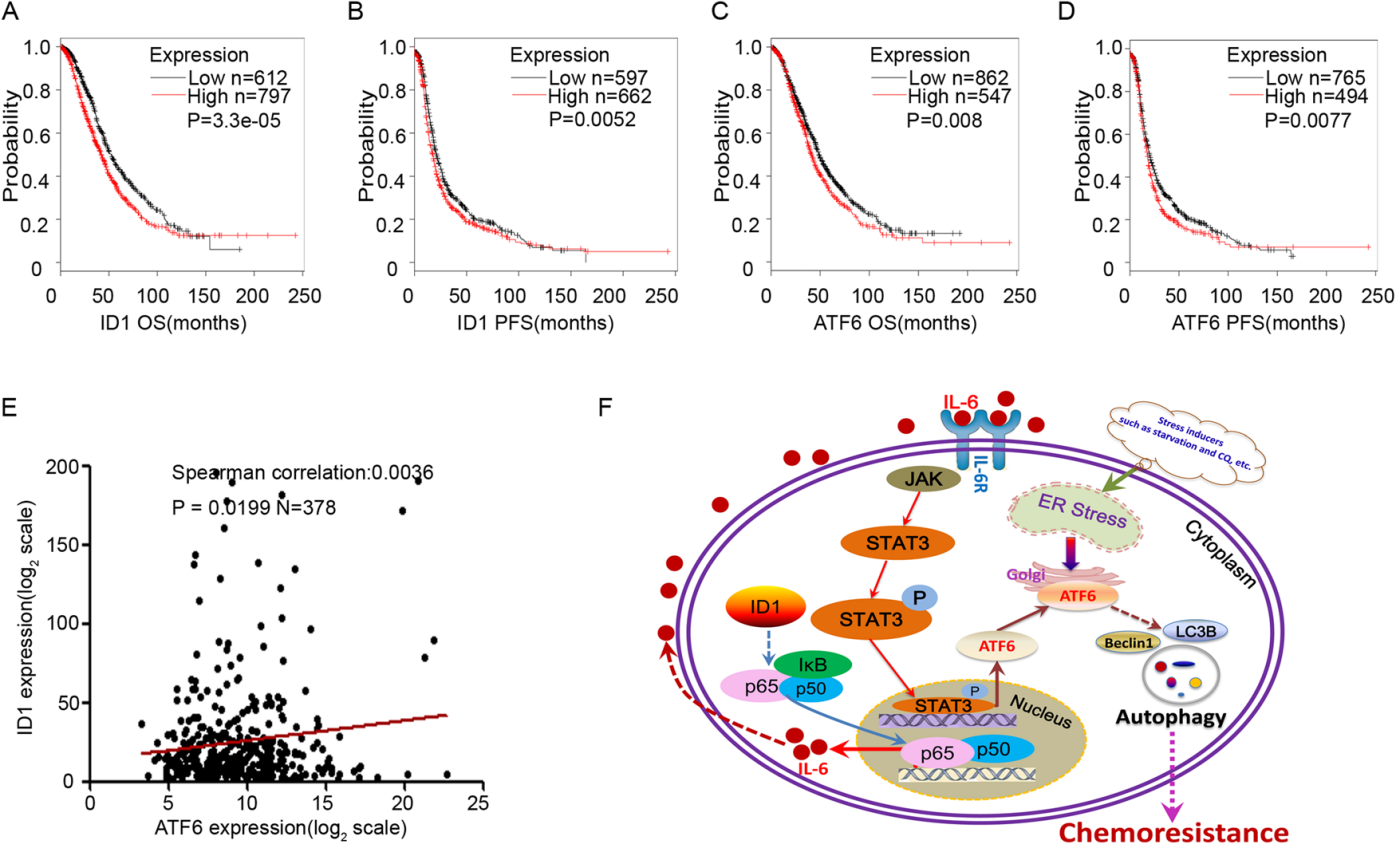
Association of ID1 and ATF6 mRNA expression with prognosis of ovarian cancer in TCGA dataset. **a**, **b** High expression of ID1 mRNA was significantly correlated with poor overall survival (OS) (**a**, *P* = 3.3e−05) and progression-free survival (PFS) (*P* = 0.0052) in patients with platinum treatment. **c**, **d** High ATF6 expression was significantly correlated with OS (P < 0.01). **d** With the probe 203952_at, elevated expression of ATF6 was related with poor PFS. Patients were treated with platinum. **e** ATF6 and ID1 are significantly correlated in ovarian cancer tissues (*P* < 0.05). **f** A schematic model showing the role of ID1, STAT3, and ATF6 in regulation of autophagy and chemoresistance. Bars represent SD from three independent experiments. All error bars = 95% CIs. *P < 0.05, **P < 0.01, ***P < 0.001.

The data from TCGA database indicated a positive correlation between ID1 and ATF6 in tissues from ovarian cancer patients treated with platinum. In patients treated with platinum, the high expression of ID1 mRNA detected with the probe 208937_s-at was significantly correlated with both short OS and progression-free survival (PFS) (*P* < 0.01). Besides, the elevated expression of ATF6 mRNA detected with the probe 203952_at was also correlated with short OS and PFS in patients treated with platinum (*P* < 0.01) (Fig. 7a–d). A significant correlation was found between ID1 and ATF6 expression (*P* < 0.05) (Fig. 7e).

## Discussion

Since ID1 is a regulator of transcription, it may be responsible for the regulation of gene expression involving multiple signal pathways. Studies have shown that ID1 regulates multiple cell processes, including proliferation, senescence, differentiation, apoptosis, and angiogenesis in various tumors^37–39^. In this report, we have identified that ID1 confers ovarian cancer cell chemoresistance through the ATF6-mediated induction of autophagy, which is a novel discovery in our study.

The mechanistic studies first showed that ID1 increased the nuclear accumulation of NF-κB p65 and decreased the cytosolic level of NF-κB p65, indicating that ID1 could enhance the overall transcription activity of NF-κB p65, which is consistent with the previous report^29^. Subsequently, we found that the expression of IL-6 was enhanced by NF-κB activation. Since the secretion of IL-6 can activate the STAT3 signaling through autocrine, we latter showed that overexpression of ID1 indeed activated the STAT3 signal pathway through the phosphorylation of STAT3 at Tyr705. Studies have implicated that the STAT3 phosphorylation at Tyr705 is involved in several steps of autophagy, from the autophagosome assembly to maturation^40,41^. Autophagy is generally held to be a constitutive process that is strongly induced during starvation^42^. Recent data indicate that ER stress and autophagy are closely associated with each other, whereas ER stress induces autophagy response through unfolded protein responses and calcium^43,44^. Our data strongly demonstrated that the ER stress-associated proteins including p4E-BP1 and ATF6 were markedly regulated by the altered gene expression of ID1 and the compound chloroquine, and that stimuli such as starvation induce autophagy development. ATF6, a type 2 transmembrane protein and an important factor in autophagy, can be induced by ER stress^45^. We identified that STAT3 is able to bind to the ATF6 promoter to promote the transcription and subsequently to induce autophagy, which is anther novel finding in this study (Fig. 7f).

Paclitaxel and cisplatin are the first-line chemotherapeutic agents used to treat ovarian cancer. However, resistance to paclitaxel and cisplatin has been a major clinical problem in ovarian cancer treatment. It is reported that the induction of autophagy may contribute to chemoresistance in lung cancer cell lines^46^ and that autophagy can promote chemoresistance in ovarian cancer^17^. We report here that overexpression of ID1 and ATF6 confers the chemoresistance to paclitaxel or cisplatin. Cells with high expression of ID1 and ATF6 appeared with higher IC50 values of cispaltin and taxol, indicating that both molecules could be targeted to improve the treatment efficacy of ovarian cancer. In our study, some important discoveries such as the cleavage of ATF6 and the feedback regulation of ID1 and STAT3 by ATF6 may need more investigations.

## Conclusions

In summary, by in vitro and in vivo experiments, we demonstrate that ID1 induces autophagy and chemoresistance through the STAT3/ATF6-mediated signal pathway. ID1, STAT3, and ATF6 may be targeted in combination with chemotherapy for ovarian cancer treatment.

## Supplementary information

Supplemental Figure 1

Supplemental Figure legend

## Data Availability

The dataset supporting the conclusions of this article is included within the article.
