# Supplementary figures and images for: ID1 confers cancer cell chemoresistance through STAT3/ATF6-mediated induction of autophagy
Source: Cell Death Dis. 2020 Feb 20;11(2):137. doi: 10.1038/s41419-020-2327-1 (PMC7033197; doi:10.1038/s41419-020-2327-1)

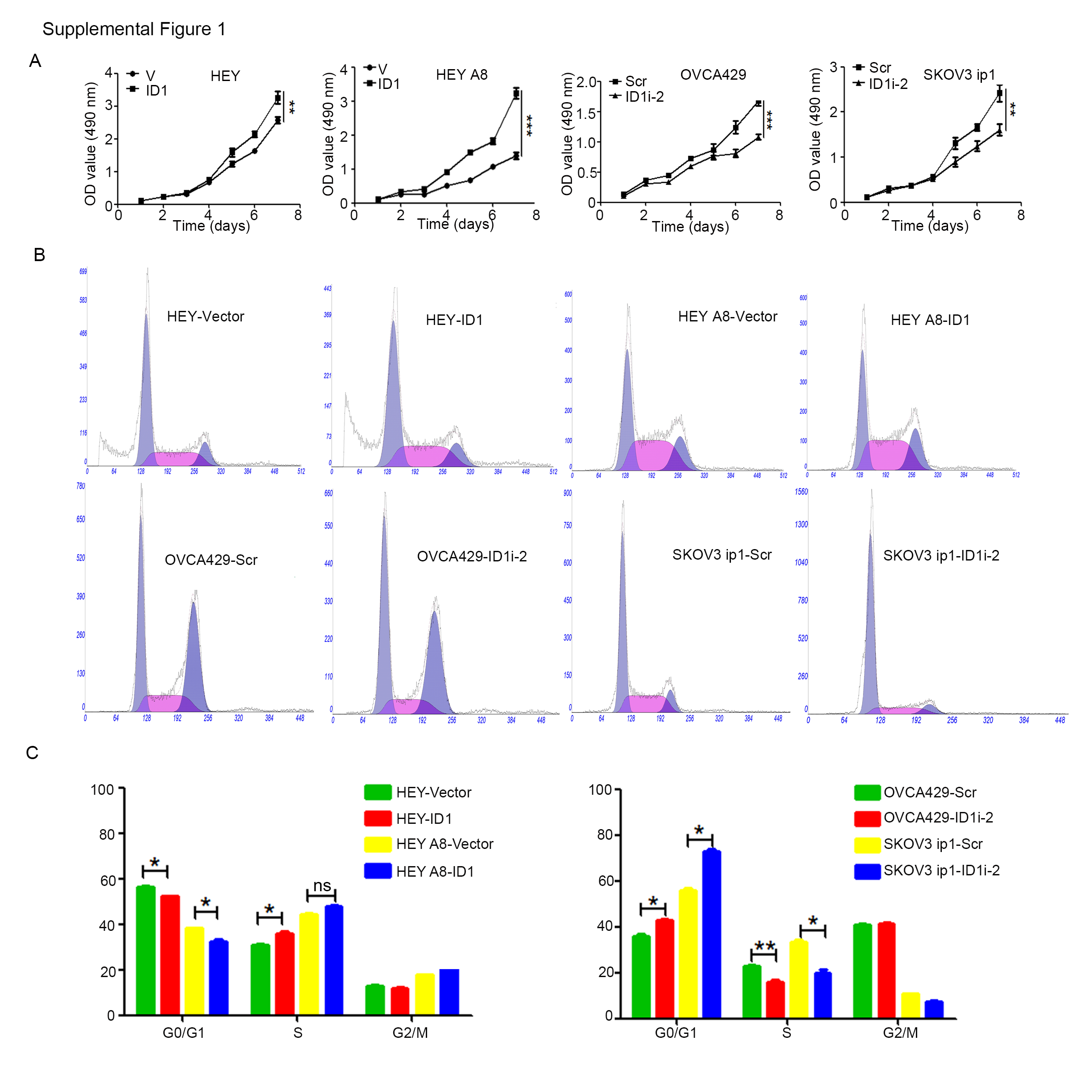

Supplement: Supplementary file 1 — Supplemental Figure 1 [file 41419_2020_2327_MOESM1_ESM.tif]
