# Supplementary material for: ID1 confers cancer cell chemoresistance through STAT3/ATF6-mediated induction of autophagy
Source: Cell Death Dis. 2020 Feb 20;11(2):137. doi: 10.1038/s41419-020-2327-1 (PMC7033197; doi:10.1038/s41419-020-2327-1)
Supplement: Supplementary file 2 — Supplemental Figure legend [file 41419_2020_2327_MOESM2_ESM.docx]

**Supplemental Figure 1. ID1 promotes cell proliferation and cell cycle progression**

**A.** Overexpression of ID1 promotes cell proliferation, but knockdown of ID1 restrains cell growth. **B.** Overexpression of ID1 induces cell cycle progression, but silencing of ID1 stimulates cell cycle arrest in ovarian cancer cells. The alteration of cell cycle was detected by Flow cytometry. **C.** Quantitative analysis of cell population at G0/G1, S and G2/M phases in cells expressing ID1 cDNA, shRNA, control vectors or scrambled shRNA by Multcycle AV DNA Analysis software equipped in Beckman flow cytometer. Bars represent SD from three independent experiments. All error bars = 95% CIs. **P* < 0.05, ** *P* < 0.01, *** *P* < 0.001, ns means *P>*0.05.
